# MicroRNA-19b-3p promotes cell proliferation and osteogenic differentiation of BMSCs by interacting with lncRNA H19

**DOI:** 10.1186/s12881-020-0948-y

**Published:** 2020-01-09

**Authors:** Gan Xiaoling, Liu Shuaibin, Liang Kailu

**Affiliations:** 1 Department of Obstetrics and Gynecology, The Second Affiliated Hospital of Chongqing Medical University, Chongqing, 400016 China; 2 Department of Orthopedics, The Second Affiliated Hospital of Chongqing Medical University, Chongqing, 400016 China

**Keywords:** miR-19b-3p, lncRNA H19, Osteoporosis, Postmenopausal, BMSC

## Abstract

**Background:**

To investigated the role of miR-19b-3p in regulating bone marrow mesenchymal stem cell (BMSC) proliferation and osteoblast differentiation.

**Methods:**

The expression of miR-19b-3p and lncRNA H19 were measured in postmenopausal osteoporosis patients and BMP-22 induced BMSCs using qRT-PCR. MiR-19b-3p mimic or inhibitor was transfected into BMP-2 induced BMSCs. Cell proliferation was measured by BrdU method. Protein expression of RUNX2 and COL1A1 were measured by western blot. PcDNA3.1-lncRNA H19 with or without miR-19b-3p mimic was transfected into BMP-2 induced BMSCs.

**Results:**

The expression of miR-19b-3p was significantly up-regulated in postmenopausal osteoporosis patients and BMP-2 induced BMSCs. MiR-19b-3p overexpression dramatically elevated, while miR-19b-3p inhibition decreased cell proliferation of BMSCs. Additionally, protein expression levels of RUNX2 and COL1A1, as well as ALP activity were significantly promoted by miR-19b-3p mimic transfection and inhibited by miR-19b-3p inhibitor transfection. LncRNA H19 was obviously down-regulated in postmenopausal osteoporosis patients. H19 overexpression significantly decreased cell proliferation and differentiation by down-regulating miR-19b-3p. Moreover, the expression of miR-19b-3p was inhibited, while H19 elvated in 17β-estradiol (E2) treated BMSCs in a dose-dependent manner.

**Conclusion:**

These data were the first to reveal the critical role of H19/miR-19b-3p in postmenopausal osteoporosis, and provided a new therapeutic target for OP.

## Background

Osteoporosis (OP) is one of the most common bone diseases worldwide, which causes increasing mortality, lasting disability and poor quality of life [1]. It is characterized by bone loss and low bone quality, which leads to fragility and fractures [2]. Osteoporosis is related to a number of risk factors, including inflammation, mechanical stress, nutrition, hormone fluctuation [3]. Bone is one of the most critical target organs of estrogen. Postmenopausal osteoporosis (PMOP), which is caused by estrogen decrease, is a major reason for bone destruction in postmenopausal women, and has become a serious threat to postmenopausal women as the world’s population ages [4]. The current treatment for postmenopausal osteoporosis are limited and often combined with a variety of complications [5]. Thus, it is urgent to explore new methods and therapeutic targets for postmenopausal osteoporosis.

Bone marrow mesenchymal stem cells (BMSCs) are source cells of osteoblasts, which are vital in maintaining the balance between bone resorption and formation [6]. It has been reported that cell proliferation and osteogenic differentiation of BMSCs are associated with osteoporosis [7]. Additionally, studies in postmenopausal osteoporosis indicated that estrogen influenced cellular process of BMSCs [8]. These findings implied a vital role of BMSCs in the development of postmenopausal osteoporosis.

MicroRNAs (miRNA) are noncoding, small RNAs that participate in regulating many diseases including osteoporosis. Accumulating evidence have shown that miRNAs regulate bone formation and resorption process, bone cell proliferation, differentiation and other functions [9]. MiR-19b-3p has been proven to regulate cellular process in the development of heart injury, cancer, osteoarthritis [10, 11]. A recent study indicated a connection between estrogen and miR-19 in chronic inflammatory diseases [12]. Thus, we suspect a role of miR-19b-3p in regulating postmenopausal osteoporosis.

In the present study, the expression of miR-19b-3p in postmenopausal osteoporosis patients and BMSCs were evaluated. Then we defined the biological effect of miR-19b-3p in regulating cell proliferation and differentiation of BMSCs. Moreover, we explored the potential correlation of lncRNA H19 and miR-19b-3p in postmenopausal osteoporosis. Therefore, we explored the role and underlying mechanism of miR-19b-3p in postmenopausal osteoporosis.

## Methods

### Subjects and specimen collection

All 18 female postmenopausal osteoporosis patients (ages 48–60 years) and 12 postmenopausal healthy controls (aged 46–58 years) were recruited from the Second Affiliated Hospital of Chongqing Medical University. 15 mL blood sample from all participate were collected. The study protocol was approved by the Ethics Committee of the Second Affiliated Hospital of Chongqing Medical University, and informed consent was obtained from all participate.

### Quantitative real-time polymerase chain reaction

Total RNA was extracted from the blood samples and cultured cells using Trizol Reagent according to the manufacturer’s protocols. The purity and concentration of RNA were detected using a NanoDrop 2000 spectrophotometer (Thermo Fisher Scientific, Shanghai, China). The cDNA was synthesized using a PrimeScript TM RT Reagent Kit (Takara Biotechnology, Dalian, China). In brief, a qRT-PCR mixture containing cDNA, primers, and SYBR-Green qPCR Master Mix was subjected to qRT-PCR quantification using an ABI Prism 7500 Sequence Detection system (Applied Biosystems, Foster City, USA). After normalization to U6 small nuclear RNA, relative lncRNA or miRNA expression was assessed.

RT-PCR.

### Cell culture

Human BMSC was purchased from American Type Culture Collection (ATCC® PCS-500-012™, ATCC, Manassas, VA). Cells were cultured in BMSC culture medium containing low-glucose Dulbecco’s modified Eagle’s medium (DMEM) supplemented with 10% fetal bovine serum (FBS). For osteoblastic differentiation, cells were cultured in medium containing 100 ng/mL BMP-2 (Gibco, Rockville, MD, USA). To evaluate the function of miR-19b-3p, cells were transfected with miR-19b-3p mimic, miR-19b-3p inhibitor, mimic control or inhibitor control (Sangon, Shanghai, China) using Lipofectamine 3000 (Invitrogen, Carlsbad, CA, USA). To alter H19 expression, cells were transfected with pcDNA3.1-H19 (Sangon, Shanghai, China), pcDNA3.1 or co-transfected with pcDNA3.1-H19 and miR-19b-3p mimic. For estrogen treatment, cells were treated with medium containing different concentrations (10^− 9^, 10^− 7^ and 10^− 5^ mol/L) of 17β-estradiol (E2) for 24 h.

### BrdU cell proliferation assay

Cell proliferation was detected using the 5-Bromo-2-deoxyUridine (BrdU) method. Briefly, cells were seeded in 96-well plates, and cultured with medium containing 100 ng/mL BMP-2. After 48 h of cell transfection, 10 μL of BrdU was added to each well and incubated for 4 h. Then, cells were fixed and incubated with BrdU antibody for 1 h. After washing three times, 200 μL of substrate solution was added to each well and incubated for 25 min, and H_2_SO_4_ was added. Finally, absorbance at 450 nm was measured using a spectrophotometer (Bio-Rad Laboratories, Hercules, CA, USA). The measured absorbance was used to calculate the cell proliferation rate.

### ALP activity

Cells were lysed using 1% Triton X-100 and then centrifuged at 10,000 rpm/min for 10 min. The supernatant was used to analyze ALP activity using an ALP Assay Kit (Sigma, St. Louis, USA). The absorbance of the reaction solution was determined at 405 nm and ALP activity was calculated according to the absorbance.

### Western blot

Total proteins were extracted from cells using ice-cold RIPA buffer. The bicinchoninic acid assay was performed to determine the protein concentration. Equal amounts of proteins (20 μg) were separated by sodium dodecyl sulfate polyacrylamide gel electrophoresis (SDS-PAGE), transferred to a polyvinylidene fluoride membrane (Bio-Rad Laboratories), and blocked in 5% non-fat milk for 1 h, followed by incubation with primary antibodies against RUNX2, COL1A1 and GAPDH (Santa Cruz Biotechnology, Santa Cruz, CA, USA) at 4 °C overnight. After three 10-min washes in phosphate-buffered saline, the membranes were incubated with a secondary antibody at room temperature for 1 h. The protein bands were visualized by enhanced chemiluminescence detection and analyzed by Image-Pro Plus software. The relative protein expression was normalized to GAPDH.

### Statistical analysis

Statistical analyses were conducted using SPSS 22.0 software. Data are presented as the mean ± standard deviation of three experiments. Differences between groups of two were evaluated by *t* test. Differences between larger groups were analyzed by one-way analysis of variance, followed by Dunnett’s test. *P* values less than 0.05 were considered significant.

## Results

### MiR-19b-3p is up-regulated in postmenopausal osteoporosis patients and BMP-2-induced BMSCs

**Fig. 1:**
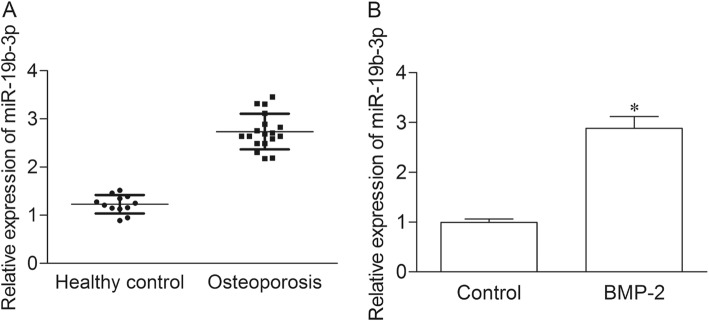
MiR-19b-3p is up-regulated in postmenopausal osteoporosis patients and BMP-2-induced BMSCs. (**a**) The expression of miR-19b-3p in the serum of postmenopausal osteoporosis patients and heathy controls were measured by qRT-PCR. Each specimen was repeated three times. (**b**) Control group, normal BMSC cell; BMP-2 group, BMSC cell treated with 100 ng/mL BMP-2. *P < 0.05 versus healthy control group

The expression of miR-19b-3p was first evaluated in the serum of postmenopausal osteoporosis patients and heathy controls by qRT-PCR. As shown in Fig. 1a, the expression of miR-19b-3p was obviously elevated in osteoporosis group as compared with healthy control group (*P* < 0.05). To explore the potential role of miR-19b-3p during osteoblast differentiation, the expression of miR-19b-3p was measured in BMSC stimulated with BMP-2, which has been proved to induce osteoblast differentiation [13]. The results indicated miR-19b-3p was significantly increased in BMP-2 induced MSCs as compared with control cells.

### MiR-19b-3p promotes proliferation of BMSCs

**Fig. 2:**
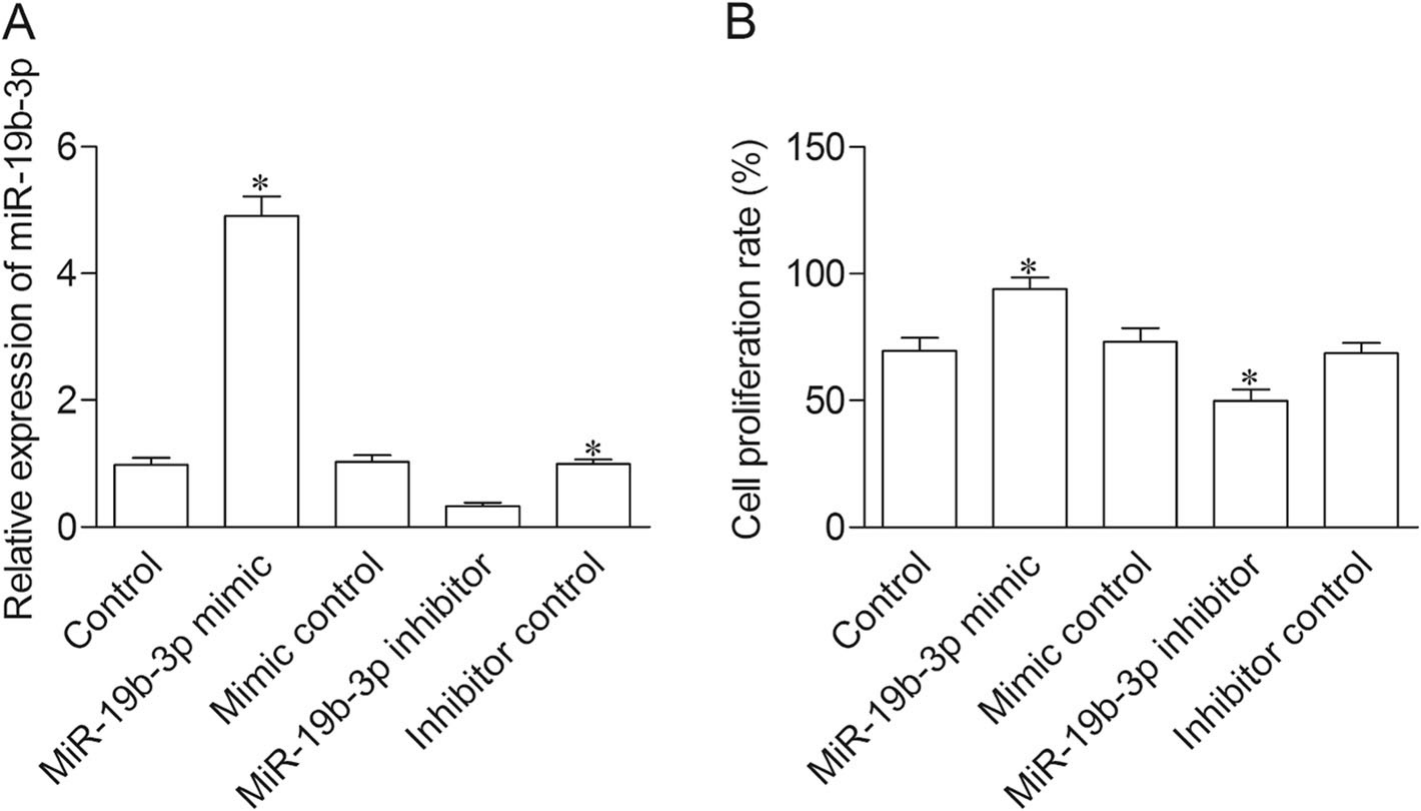
MiR-19b-3p promotes proliferation of BMSCs. Control group, BMSC cells treated with BMP-2; miR-19b-3p mimic group, BMP-2 treated cells transfected with miR-19b-3p mimic; mimic control group, BMP-2 treated cells transfected with mimic control; miR-19b-3p inhibitor group, BMP-2 treated cells transfected with miR-19b-3p inhibitor; inhibitor control group, BMP-2 treated cells transfected with inhibitor control. (**a**) The expression of miR-19b-3p was measure by qRT-PCR. (**b**) Cell proliferation rate was evaluated by BrdU assay. *P < 0.05 versus healthy control group

To determine the effect of miR-19b-3p on cell proliferation, miR-19b-3p mimic or inhibitor was transfected into BMP-2 induced BMSCs. The qRT-PCR results showed a significant increase of miR-19b-3p expression in miR-19b-3p mimic transfection group, and an obvious decrease of miR-19b-3p expression in miR-19b-3p inhibitor transfection group as compared with control group (Fig. 2a). BrdU results indicated that cell proliferation level was significantly elevated in miR-19b-3p mimic group, while dramatically declined in miR-19b-3p inhibitor group as compared with control group (Fig. 2b).

### MiR-19b-3p boost differentiation of BMSCs

**Fig. 3:**
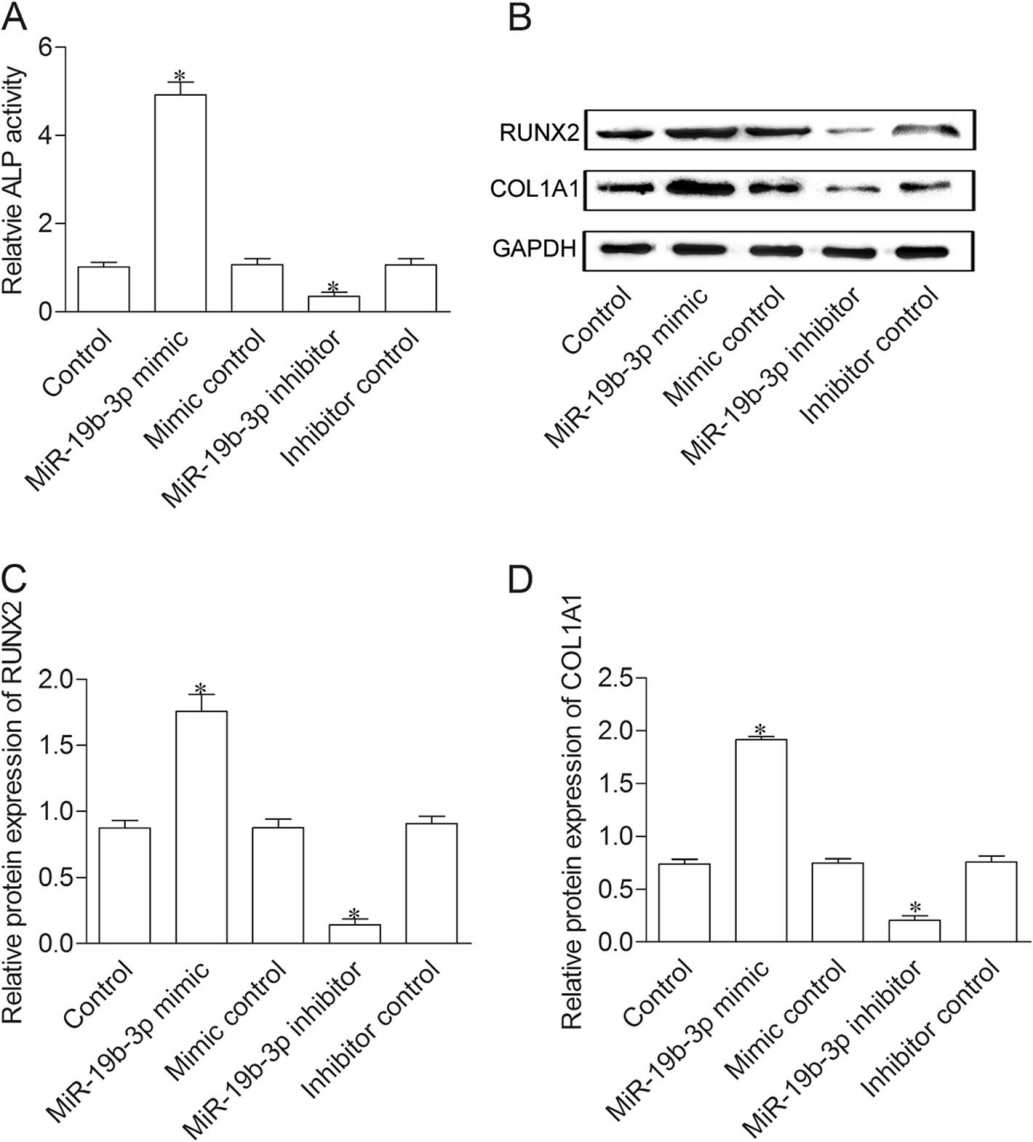
MiR-19b-3p boost differentiation of BMSCs. (**a**) ALP activity was detected in the supernatant of cells. (**b**) Protein expression of RUNX2 and COL1A1 were measured by western blot method. (**c** and **d**) Relative protein level was normalized to GAPDH. *P < 0.05 versus healthy control group

To evaluate the effect of miR-19b-3p on BMSC differentiation, we measured ALP activity and the expression level of RUNX2, COL1A1 in BMP-2 induced BMSCs. As showed in Fig. 3a, ALP activity was significantly elevated in miR-19b-3p mimic group, while decreased in miR-19b-3p inhibitor group as compared with control group. In addition, protein expression of RUNX2 and COL1A1 were enhanced in miR-19b-3p mimic group, whereas impeded in miR-19b-3p inhibitor group compared to control group (Fig. 3b, c and d).

### H19 up-regulation elevates cell proliferation and differentiation of BMSCs through mediating miR-19b-3p

**Fig. 4:**
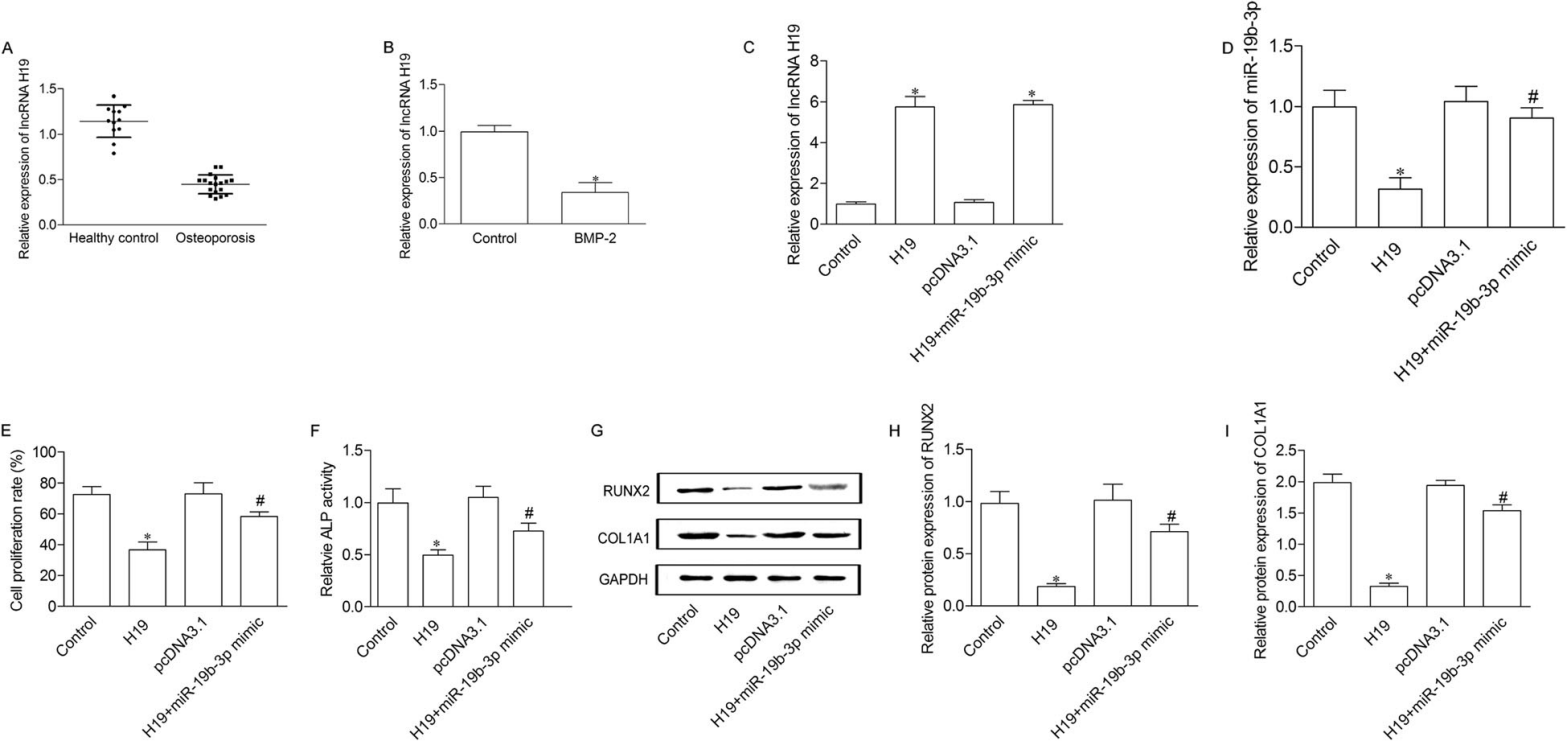
H19 up-regulation elevates cell proliferation and differentiation of BMSCs through mediating miR-19b-3p. (**a**) The expression of H19 in the serum of postmenopausal osteoporosis patients and heathy controls were measured by qRT-PCR. Each specimen was repeated three times. (**b**) Control group, normal BMSC cell; BMP-2 group, BMSC cell treated with 100 ng/mL BMP-2. *P < 0.05 versus healthy control group. Control group, BMSC cells treated with BMP-2; H19 group, BMP-2 treated cells transfected with pcDNA3.1-H19; pcDNA3.1 group, BMP-2 treated cells transfected with pcDNA3.1 empty vector; H19 + miR-19b-3p mimic, BMP-2 treated cells co-transfected with pcDNA3.1-H19 and miR-19b-3p mimic. H19 (**c**) and miR-19b-3p (**d**) expression were measured by qRT-PCR. (**e**) Cell proliferation was measured by BrdU assay. (**f**) ALP activity was detected in the supernatant of cells. (**g**) Protein expression of RUNX2 and COL1A1 were measured by western blot method. (**h** and **i**) Relative protein level was normalized to GAPDH. *P < 0.05 versus healthy control group. *P < 0.05 versus control group; #*P* < 0.05 versus H19 group

H19 expression was determined in postmenopausal osteoporosis patients and healthy controls. The results showed a significant decrease of H19 expression in postmenopausal osteoporosis patients compared to healthy controls (Fig. 4a). We then evaluated the expression of H19 in BMP-2 stimulated BMSCs. The results indicated H19 was significantly decreased in BMP-2 induced BMSCs compared to control cells (Fig. 4b). To investigate the correlation between H19 and miR-19b-3p in BMSCs, we transfected pcDNA3.1-H19 alone or with miR-19b-3p mimic into BMP-2 induced BMSCs. As indicated in Fig. 4c, H19 expression was significantly enhanced in H19 group as compared with control group. Meanwhile, there is no significant difference between H19 group and H19 + miR-19b-3p mimic group. Additionally, miR-19b-3p expression was significantly down-regulated in H19 group compared to control group, and increased in H19 + miR-19b-3p mimic group compared to H19 group (Fig. 4d). Cell proliferation was decreased in pcDNA3.1-H19 transfected BMSCs, and elevated after adding miR-19b-3p mimic (Fig. 4e). Moreover, ALP activity (Fig. 4f) as well as protein expression of RUNX2 and COL1A1 (Fig. 4g, h and i) were decreased in H19 group compared to control group, while significantly increased in H19 + miR-19b-3p group compared to H19 group.

### Estrogen down-regulated miR-19b-3p and up-regulated H19 in BMSCs

**Fig. 5:**
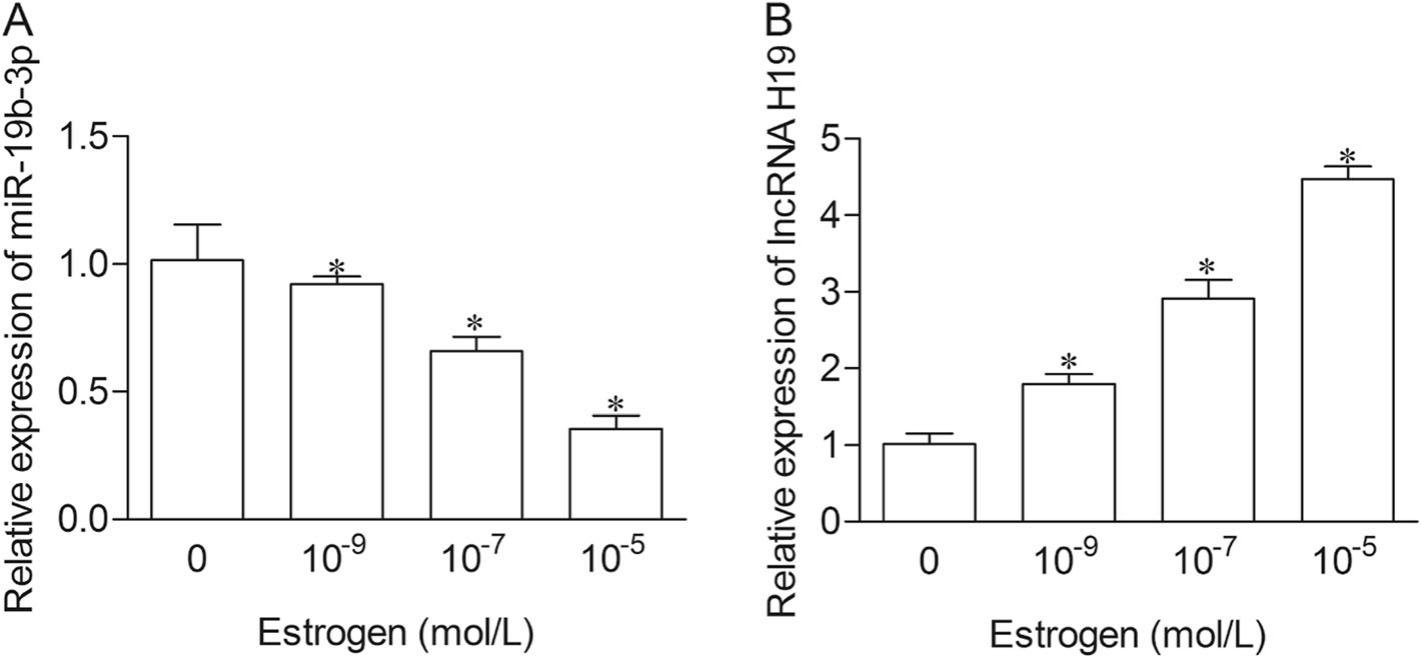
Estrogen down-regulated miR-19b-3p and up-regulated H19 in BMSCs. BMSCs were treated with different concentration of E2 (10^− 9^, 10^− 7^ and 10^− 5^ mol/L). The expression of miR-19b-3p (**a**) and H19 (**b**) were measured by qRT-PCR. *P < 0.05 versus control group

To further explore the role of H19/miR-19b-3p in postmenopausal osteoporosis, BMSCs were cultured with different concentrations of E2. The expression level of miR-19b-3p expression was dramatically impeded, while H19 was significantly elevated in a dose-dependent manner in estrogen cultured groups (Fig. 5a and b).

## Discussion

Postmenopausal osteoporosis is a relatively silent disease with no symptoms until fractures take place [14]. The occurrence and development of PMOP was closely associated with the cellular state of BMSCs [15]. Nowadays, studies have elucidated than miRNAs can regulate cell apoptosis, proliferation, differentiation and other cellular process [16]. This research was the first to demonstrate the role and underlying mechanism of miR-19b-3p in regulating cell proliferation and differentiation of BMSCs in postmenopausal osteoporosis.

MiR-19b is part of the miR-17-92 cluster which encodes miR-17, miR-18a, miR-19a, miR-19b, miR-20a and miR-92a [17]. A number of studies have reported that the miR-17-92 cluster regulated cellular process in various cancers, immune diseases, heart conditions and other dysfunction [18]. A previous study has reported that miR-17-5p modulated osteoblastic differentiation and cell proliferation in non-traumatic osteoporosis [19]. Another study showed that estrogen modulates inflammatory response of monocytes thorough elevating miR-19b [12]. To our knowledge, no study has addressed the role of miR-19b-3p in postmenopausal osteoporosis. We therefore measured the expression of miR-19b-3p in PMOP patients and found it to be significantly increased compared with heathy control subjects. However, it should be emphasized that our patients sample size is small, and further study with larger cohorts was needed to verify the specificity of this miRNA. The cytokine BMP-2 has been proved to boost osteoblastic differentiation of BMSCs [20]. Our data showed an obvious increase of miR-19b-3p expression in BMP-2 induced BMSCs, suggested a connection between miR-19b-3p and osteoblastic differentiation, and further implied the biomarker potential of miR-19b-3p in PMOP.

The cellular function of BMSCs is vital in the progress of PMOP. Differentiation of BMSCs into osteoblasts is of great importance in maintaining normal bone mineral density and adjusting bone formation [21]. In our study, we observed up-regulation of miR-19b-3p promoted, while down-regulation of miR-19b-3p inhibited cell proliferation of BMSCs. The effects of miR-19b-3p in osteogenic differentiation markers, including and ALP, were also determined [22]. The data confirmed miR-19b-3p was a positive regulator for osteogenic differentiation.

It is well known that changes in molecular expression are usually regulated by upstream molecules. LncRNAs are a type of non-coding RNAs, which contains over 200 nucleotides [23]. Growing research has demonstrated that lncRNA modulates gene expression at multiple levels such as epigenetic, transcriptional and post transcriptional [24]. LncRNAs could act as competitive endogenous RNAs and regulate the expression and activity of miRNAs [24]. LncRNA H19 has been addressed in various cancers as an oncogene, and regulated cell proliferation, apoptosis and migration [25]. It has been found that H19 down-regulation modulated osteogenic differentiation of BMSCs from ovariectomized mouse, which suggested an important role of H19 in postmenopausal osteoporosis [26]. In the present study, we observed a significant decrease of H19 expression in postmenopausal osteoporosis patients and in BMP-2 induced BMSCs compared with controls, which was consistent with a former study [26]. And more importantly, our results indicated that H19 overexpression down-regulated miR-19b-3p expression in BMSCs. Our further experiments demonstrated that the decrease of cell proliferation and osteogenic differentiation induced by H19 up-regulation was reversed by miR-19b-3p mimic. These data indicated that H19 was involved in the regulation of BMSCs through modulating miR-19b-3p.

Estrogen decrease is an important cause of osteoporosis in women [27]. This Study have shown that estrogen deficiency induced bone loss and structural degeneration [28]. Additionally, estrogen influence expression of molecular regulators of BMSCs, and affects the development of osteoporosis [29]. The data in our study indicated that estrogen inhibited miR-19b-3p, while elevated H19 expression in a dose-dependent manner, suggested a clear connection between estrogen and H19/miR-19b-3p, further confirm the biomarker potential of H19 and miR-19b-3p in postmenopausal osteoporosis.

## Conclusions

Our study, for the first time, indicated important role of miR-19b-3p in regulating proliferation and osteogenic differentiation of BMSCs in postmenopausal osteoporosis. It further revealed the mechanistic evidence of H19 down-regulation and the resulting promotion of miR-19b-3p in regulating BMSCs. This study can expand a new way of preventing postmenopausal osteoporosis and provide a theoretical basis for developing new treatment of targeting miR-19b-3p.

## Data Availability

The datasets used and/or analyzed during the current study are available from the corresponding author on reasonable request.

## Abbreviations

BMSC: Bone marrow mesenchymal stem cell
DMEM: Dulbecco’s modified eagle’s medium
E2: 17β-estradiol
FBS: Fetal bovine serum
miRNA: MicroRNAs
OP: Osteoporosis
PMOP: Postmenopausal osteoporosis
